# Multilevel Approach for the Treatment of Giardiasis by Targeting Arginine Deiminase

**DOI:** 10.3390/ijms22179491

**Published:** 2021-08-31

**Authors:** Cynthia Fernández-Lainez, Ignacio de la Mora-de la Mora, Itzhel García-Torres, Sergio Enríquez-Flores, Luis A. Flores-López, Pedro Gutiérrez-Castrellón, Lilian Yépez-Mulia, Felix Matadamas-Martínez, Paul de Vos, Gabriel López-Velázquez

**Affiliations:** 1 Laboratorio de Errores Innatos del Metabolismo y Tamiz, Instituto Nacional de Pediatría, Ciudad de Mexico 04530, Mexico; lainezcynthia@hotmail.com; 2 Immunoendocrinology, Division of Medical Biology, Department of Pathology and Medical Biology, University of Groningen and University Medical Centre Groningen, Hanzeplein 1, 9700 RB Groningen, The Netherlands; p.de.vos@umcg.nl; 3 Posgrado en Ciencias Biológicas, Universidad Nacional Autónoma de México, Ciudad de Mexico 04510, Mexico; 4 Laboratorio de Biomoléculas y Salud Infantil, Instituto Nacional de Pediatría, Ciudad de Mexico 04530, Mexico; ignaciodelamora@yahoo.com.mx (I.d.l.M.-d.l.M.); itzheltorres@hotmail.com (I.G.-T.); sergioenriquez@ciencias.unam.mx (S.E.-F.); luisbiolexp@gmail.com (L.A.F.-L.); 5 CONACYT-Instituto Nacional de Pediatría, Secretaría de Salud, Ciudad de Mexico 04530, Mexico; 6 Hospital General Dr. Manuel Gea González, Ciudad de Mexico 14080, Mexico; inpcochrane@gmail.com; 7 Unidad de Investigación Médica en Enfermedades Infecciosas y Parasitarias, UMAE Hospital de Pediatría, Centro Médico Siglo XXI, Instituto Mexicano del Seguro Social, Ciudad de Mexico 06720, Mexico; lilianyepez@yahoo.com (L.Y.-M.); felixmatadamas@yahoo.com.mx (F.M.-M.)

**Keywords:** *Giardia*, drug repurposing, proton pump inhibitor, sulbutiamine, aurothiomalate, omeprazole, rabeprazole

## Abstract

Giardiasis represents a latent problem in public health due to the exceptionally pathogenic strategies of the parasite *Giardia lamblia* for evading the human immune system. Strains resistant to first-line drugs are also a challenge. Therefore, new antigiardial therapies are urgently needed. Here, we tested giardial arginine deiminase (GlADI) as a target against giardiasis. GlADI belongs to an essential pathway in *Giardia* for the synthesis of ATP, which is absent in humans. In silico docking with six thiol-reactive compounds was performed; four of which are approved drugs for humans. Recombinant GlADI was used in enzyme inhibition assays, and computational in silico predictions and spectroscopic studies were applied to follow the enzyme’s structural disturbance and identify possible effective drugs. Inhibition by modification of cysteines was corroborated using Ellman’s method. The efficacy of these drugs on parasite viability was assayed on *Giardia* trophozoites, along with the inhibition of the endogenous GlADI. The most potent drug against GlADI was assayed on *Giardia* encystment. The tested drugs inhibited the recombinant GlADI by modifying its cysteines and, potentially, by altering its 3D structure. Only rabeprazole and omeprazole decreased trophozoite survival by inhibiting endogenous GlADI, while rabeprazole also decreased the *Giardia* encystment rate. These findings demonstrate the potential of GlADI as a target against giardiasis.

## 1. Introduction

Current drug discovery techniques against parasites aim at the identification of essential pathogenic targets, which, after inhibiting their functionality, could be lethal for the pathogenic organisms. In addition, anti-virulence therapy is based on disarming the pathogens and preventing them from causing disease. The latter is increasing because virulence factors enhance the ability of pathogens to colonize and resist the immunity of their hosts. Hence, therapeutic strategies directed at the molecules that participate at different levels in the life cycle of pathogens might be helpful in the treatment of the illness, but also in repressing the dissemination of the disease. Such a strategy might also be helpful in the fight against giardiasis.

Human giardiasis is an inflammation-driven diarrhea caused by the protist, *Giardia* *lamblia* [1], which has developed a broad range of mechanisms to escape the host immune system [2]. *Giardia* should not fall on deaf ears; it is a eukaryote more ancient than humans and is still epidemic, despite the tremendous advances in healthcare. *Giardia* is a microaerophilic organism that lacks the tricarboxylic acid cycle and oxidative phosphorylation, but has acquired enzymes of likely lateral gene transfer origin that support fermentative glycolysis [3], the arginine dihydrolase pathway [4,5], and substrate-level ATP generation [6,7,8].

Drug repurposing for the treatment of giardiasis based on cysteine-modification mechanisms directed at glycolysis has previously been proposed [9,10,11]. Since cysteines are highly reactive amino acids and are widely distributed in the proteome of *Giardia* [12], strategies based on the use of thiol-reactive drugs could target several important proteins of this disease that are still neglected. In this line, the short-term use of proton pump inhibitors (PPIs) is well tolerated, with few side effects [13]. Moreover, commercially available PPIs are active in vitro against *Giardia* trophozoites in the range of albendazole [14] and also retain their activity for metronidazole and nitazoxanide-resistant strains [11]. Such a landscape appears propitious for designing effective therapies based on thiol-reactive drugs against *Giardia* and for widening the spectrum of repurposable drugs for giardiasis.

In recent years, new drug targets have been studied in the search for efficient compounds against protozoa [9,15,16,17]. Based on this, we studied the giardial arginine deiminase (GlADI) as a crucial biomolecule essential for *Giardia* survival and involved in disease progression. To better support GlADI as a druggable target in *Giardia*, we studied its reactivity to s-methyl methanethiosulfonate (MMTS) and dithio-bis-nitrobenzoic acid (DTNB), to demonstrate the capacity of thiol-reactive compounds to inactivate this enzyme. Afterward, we assayed the commercially approved drugs omeprazole, rabeprazole, sulbutiamine, and aurothiomalate as repurposed drugs for giardiasis (Figure S1). Furthermore, we established the potential of these drugs for use as antigiardiasics by assaying them from a molecular perspective, using in silico studies, as well as biochemical and cellular studies. We found that rabeprazole is the most promising drug for repurposing against giardiasis, since it inactivates the enzyme activity of GlADI, disturbs its structure, provokes *Giardia* trophozoites death, and diminishes encystation.

## 2. Results

### 2.1. Cysteine Modification Underlies GlADI Inactivation

Since the complete 3D structure of GlADI is still unknown, homology modeling was used to identify potential inhibitors by docking [18]. We used the crystallographic structure of the arginine deiminase from *Mycoplasma arginini* as a template to model the GlADI 3D structure. This led to a number of observations. First, it revealed the abundance and distribution of the Cys residues in the three-dimensional arrangement of the homodimer, and second, it allowed us to identify the preferential binding sites of the highly Cys-reactive compounds MMTS and DTNB on the protein surface. After in silico approximations, the effect on the enzyme activity of MMTS and DTNB was determined in vitro, and finally, the free cysteine content was determined using Ellman’s method to establish the underlying mechanism of inactivation by modification of cysteines.

**Figure 1:**
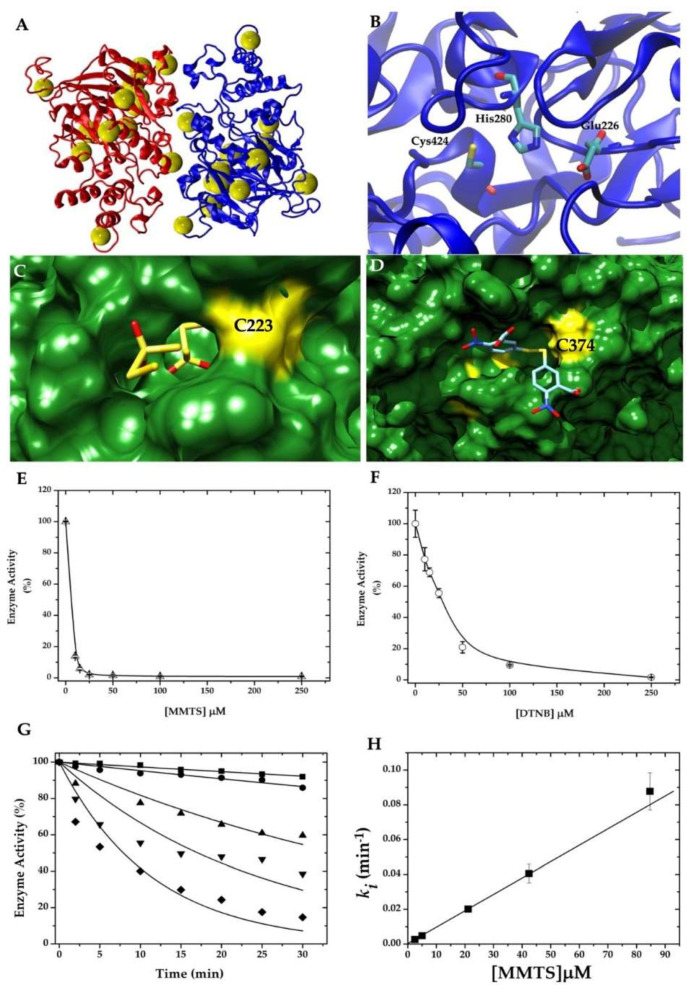
Arginine deiminase from *Giardia* as a potential target for cysteine reactive compounds. (**A**) Homology model showing the high content and distribution of cysteine residues (yellow spheres). (**B**) active site showing the catalytic triad. (**C**) representative docking with MMTS, and (**D**) with DTNB. Cysteine residues interacting with both molecules are indicated in bold type. Residual enzyme activity of recombinant GlADI after incubation with increasing concentrations of (**E**) MMTS or (**F**) DTNB. (**G**) Kinetics of GlADI inhibition by using 2. 5 (■), 5 (●), 20 (▲), 45 (▼), and 85 μM (◆) of MMTS. Inactivation rate constants at different MMTS concentrations were fitted to a linear equation model, where the slope represents the second−order inactivation rate constant (**H**).

GlADI has the longest known C-terminal domain among the described arginine deiminases. Through the dimer, 26 of the 32 Cys residues can be observed (Figure 1A and Figure S2). Of all modeled Cys residues, 13 show more than a 10% accessible surface area (ASA) (Table 1). The model was helpful in showing the distribution of the catalytic triad [19], which includes Cys 424, Glu 226, and His 280 amino acid residues (Figure 1B). The blind docking performed on the entire surface of the homodimer identified potential binding sites for MMTS and DTNB. Representative binding sites for each compound are shown in Figure 1C,D. After the in silico approximations, the effect of MMTS and DTNB on the GlADI enzyme activity was assayed in vitro. The interaction between these compounds and GlADI caused inactivation of the enzyme in a dose-dependent manner (Figure 1E,F).

Since MMTS resulted in the most efficient inhibition of GlADI (Figure 1E), we calculated its pseudo-first-order inactivation rate constant. The results show that the kinetics of inhibition using different concentrations of MMTS decays following a mono-exponential function (Figure 1G). Notwithstanding, the parameters used to adjust each data series were the same; those from 42 μM MMTS (▼) showed the lowest R^2^ value (0.74, whereas the rest of the concentrations showed R^2^ values above 0.9). This yielded an increased error for this *ki* value, but it did not prevent the linear fitting to calculate the second-order rate constant (*k*_2_). The data of the slopes fitted onto a straight-line (Figure 1H) with a *k*_2_ value of 15.68 ± 0.9 M^−1^ s^−1^.

Concerning the underlying mechanism, the observed change of free cysteines before and after the treatments supports the inactivation being caused by a Cys-modification process (Table 2).

### 2.2. GlADI Is Inactivated by Thiol-Reactive Drugs Not Previously Applied for Giardiasis

**Figure 2:**
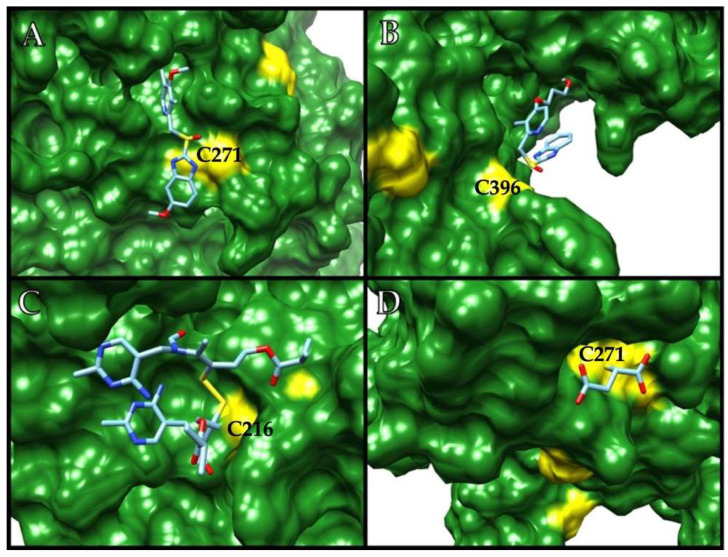
Representative docking of GlADI with (**A**) omeprazole, (**B**) rabeprazole, (**C**) sulbutiamine, and (**D**) aurothiomalate. Cysteine residues near the binding sites are yellow-colored. Cysteine residues interacting with each of the molecules are indicated in bold type.

**Figure 3:**
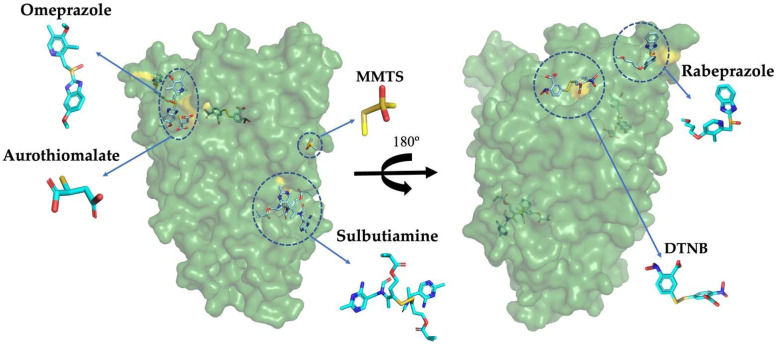
Two views of the GlADI monomer A rotated 180°, showing the global distribution of all the studied molecules in their putatively best ranked binding sites. To make the visualization easier, the structure is displayed with 50% transparency.

Based on the in vitro susceptibility of GlADI to being inactivated through a cysteine modification process, we studied four different thiol-reactive drugs: omeprazole, rabeprazole, sulbutiamine, and aurothiomalate, which are known to be safely used in humans but not for giardicidal purposes. As expected, the docking performed on the surface of the enzyme showed several possible binding sites near cysteine residues for all the studied drugs (representative binding sites for each drug are shown in Figure 2A–D). Moreover, an overall view of the GlADI monomer A shows the putative binding sites of the studied molecules on the general topography of the protein (Figure 3).

**Figure 4:**
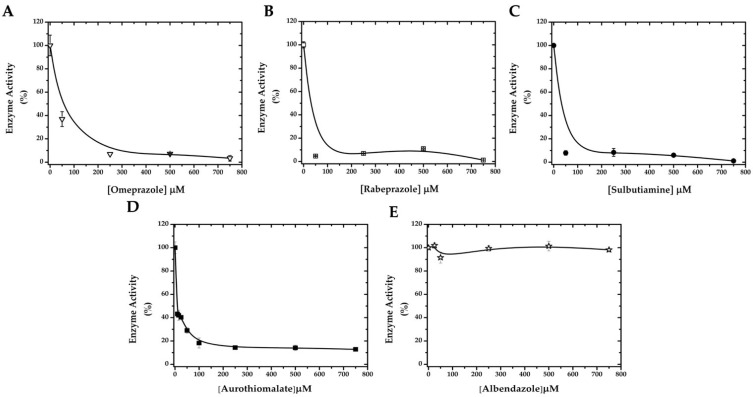
Effect of repurposing drugs approved for use in humans on the enzyme activity of GlADI. The enzyme was incubated with increasing concentrations of omeprazole (**A**), rabeprazole (**B**), sulbutiamine (**C**), aurothiomalate (**D**), and albendazole (**E**).

In agreement with the in silico prediction, all of these drugs were able to inhibit recombinant GlADI in vitro in a dose-dependent manner (Figure 4A–D). However, the number of modified cysteines on GlADI was different for each compound (Table 2). To further support Cys-modification as the underlying mechanism for GlADI inactivation, albendazole was assayed as a giardicidal but non-thiol-reactive drug. As expected, albendazole did not produce any inactivation of GlADI (Figure 4E).

**Figure 5:**
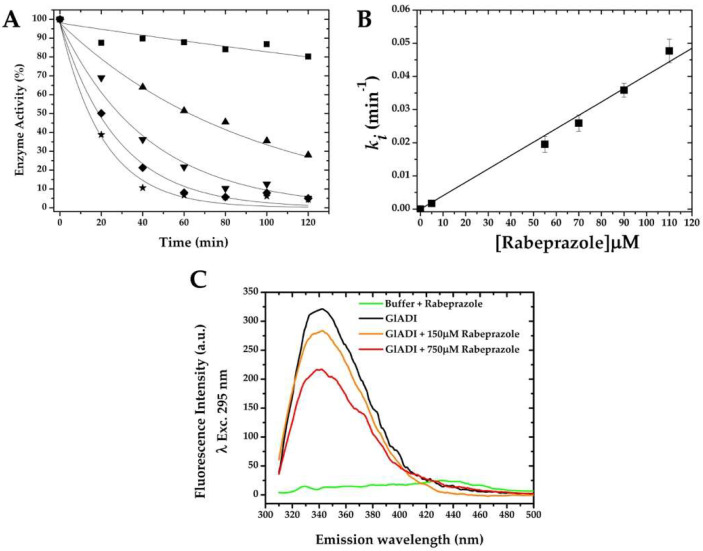
Effect of rabeprazole on the inactivation and intrinsic fluorescence of GlADI. (**A**) GlADI was incubated with 5 (■), 55 (▲), 70 (▼), 90 (◆), and 110 μM (★) of rabeprazole at 25 °C. (**B**) Inactivation rate constants at each concentration were plotted and fitted to a linear equation model, where the slope represents the second-order inactivation rate constant. (**C**) Measure of the fluorescence intensity of GlADI in the presence and absence of rabeprazole.

Since rabeprazole showed an efficient inhibition of the enzyme activity on recombinant GlADI (Figure 4B), we calculated the pseudo-first-order inactivation rate constant for this drug (Figure 5A,B). The results show that the kinetics of inhibition using different concentrations of rabeprazole decay following a mono-exponential function (Figure 5A). This data fitted onto a straight-line with a second-order rate constant (*k*_2_) value of 6.72 ± 0.2 M^−1^ s^−1^ (Figure 5B).

To quantify the potential conformational changes of GlADI 3D structure by rabeprazole, fluorescence spectroscopy at 295 nm excitation wavelength was performed to monitor tryptophan residues. The intensity of intrinsic fluorescence decreased as a result of rabeprazole concentration (Figure 5C).

### 2.3. Targetable Cysteines in GlADI Are Possibly Limited by the Presence of Disulfide Bridges in the Native Structure

The amino acid sequence of GlADI revealed the presence of 16 Cys residues per monomer. This number of Cys residues was corroborated and deduced by sequencing the gene coding the recombinant enzyme we had previously studied (data not shown). Nonetheless, the maximum number of Cys residues accessible for the Ellman’s reagent was six per monomer in the native enzyme and nine after denaturation. Conversely, when breaking all the disulfide bonds under reducing conditions, Ellman’s reagent was able to detect the 16 Cys-residues per monomer after denaturing (Table 2).

### 2.4. Drug Susceptibility of Giardia Trophozoites Discloses the Efficacy of the Proposed Drugs

**Figure 6:**
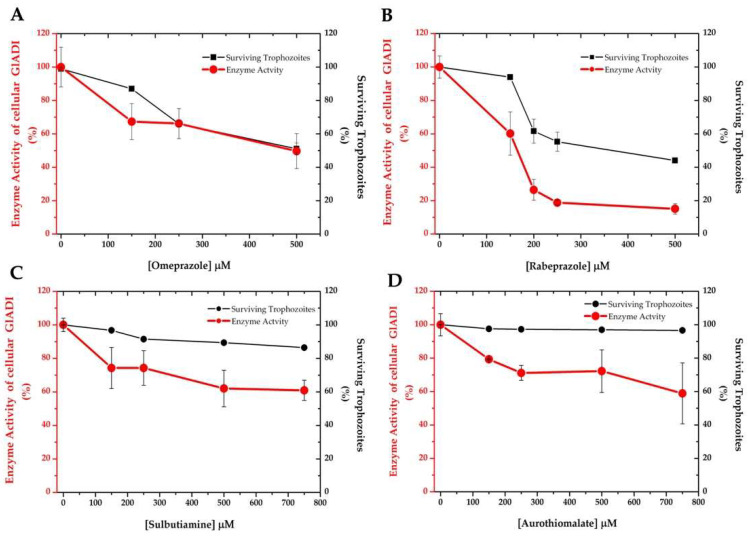
Effect of repurposing drugs on *Giardia* trophozoite survival and on the enzyme activity of endogenous GlADI. Omeprazole and rabeprazole exerted a concomitant effect on these parameters (**A**,**B**), whereas sulbutiamine and aurothiomalate did not (**C**,**D**).

Relying on druggability prediction can generate ambiguous expectations, and final effects need to be determined on a cellular level. Therefore, we also determined *Giardia* trophozoites inhibition by the four drugs we found promising in our in silico and in vitro predictions. This revealed that only the PPIs showed a dose-dependent giardicidal effect on trophozoites by inactivation of the endogenous cellular GlADI (Figure 6).

### 2.5. Rabeprazole Impairs Giardia Encystment

**Figure 7:**
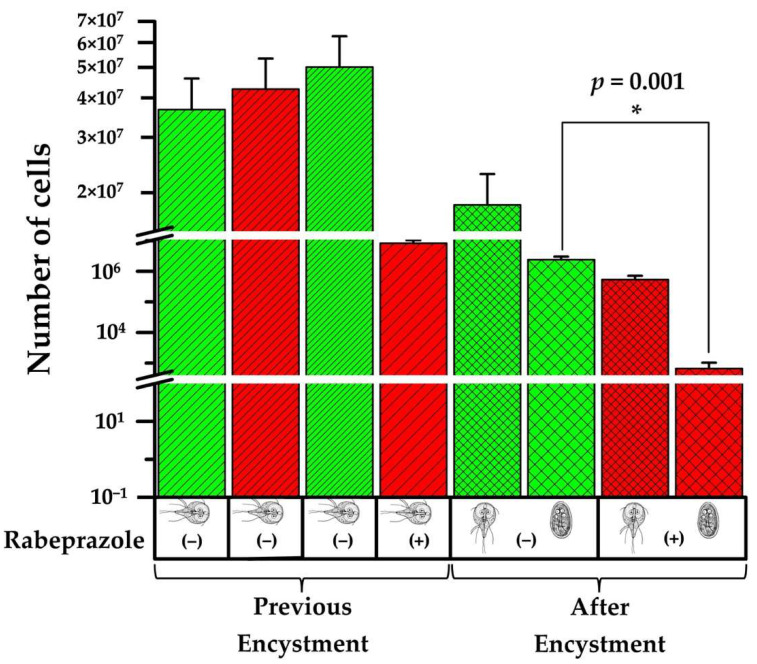
Impairment of *Giardia* encystment by rabeprazole. Green columns represent the control group (without rabeprazole), and the red columns represent the experimental group (exposed to rabeprazole). From left to right, the first two columns represent the number of cells grown to confluence at the beginning of the experiment in TYI−S−33 culture medium. The next two columns represent the number of cells grown in pre-encystment culture medium. The last four columns represent the number of cells grown in encystment culture medium. These results are the medium ± SD of three assays, with three technical replicates.

Cyst formation is a requirement for surviving environmental stresses during the transmission of *Giardia*. This infective form of the parasite represents a target for blocking its dissemination [20]. The findings shown herein prompted us to investigate the effect of rabeprazole on the encystment process of *Giardia*. As shown above, rabeprazole decreases trophozoite viability (Figure 6B). We found that it also significantly affects encystation (*p* = 0.001) (Figure 7). Contrary to what was previously proposed as the role of GlADI in the control of the encystation process [21], we found a significant decrease of cyst production between rabeprazole treated and untreated trophozoites (Figure 7). Moreover, the trophozoites that survived the treatment with rabeprazole were 97% less efficient at encysting than those not treated.

## 3. Discussion

The screening for new giardicidal drugs is undoubtedly a priority, since drug resistance and treatment failures are increasing [22]. As GlADI is absent in the human host, this enzyme is an attractive target for drug design against giardiasis [23]. Computational studies have enhanced the ability to pose reasonable and testable hypotheses for drug repositioning purposes. Therefore, druggability predictions have become part of the validation of a therapeutic target, to avoid intractable targets [24]. Nonetheless, the critical nature of these approaches is evident in cases where data gathered from in silico predictions do not ensure their efficiency in subsequent in vitro studies.

Based on computational docking, benzimidazole derivatives apart from PPIs have been proposed as potential inhibitors of GlADI [18]. Since the primary structure of GlADI is enriched with cysteine residues, we performed computational approaches to find potential inhibitors with an underlying cysteine-modification mechanism of action. With this approach, we found several thiol-reactive compounds (including some repurposing drugs) with promising binding regions on GlADI. Furthermore, all the thiol-reactive compounds studied in silico demonstrated high efficacy for inhibiting recombinant GlADI when they were assayed in vitro.

The number of modified cysteines in GlADI was variable according to the thiol-reactive compound assayed. This is possibly related to the accessibility of each cysteine residue, but it also might be because more than one Cys can contribute to the inactivation of the enzyme. It is noteworthy that the number of accessible cysteines in denatured conditions indicated the presence of at least three disulfides. Such a condition had already been reported for other giardial proteins (i.e., variable surface proteins, triosephosphate isomerase) [25,26], with structural and functional implications in some cases [27]. This should be further studied to better understand the biological meaning of these disulfides.

The calculated second-order inactivation constants (*k*_2_) were in the range of others previously determined for different giardial enzymes [9]. Indeed, the value of the *k*_2_ obtained for rabeprazole (Figure 5A,B) was 2.5-fold higher than that of rabeprazole, inhibiting the giardial triosephosphate isomerase [9], which means this drug possesses a higher efficiency for inhibiting GlADI than inhibiting triosephosphate isomerase. This would indicate that GlADI is a target more easily reached by rabeprazole than other targets previously proposed for this drug, such as triosephosphate isomerase. Therefore, since GlADI belongs to the arginine dihydrolase pathway, this route would be impaired in a more immediate manner than glycolysis.

It is known that thiol-reactive drugs do not reach all Cys present in a protein and, even if a Cys is modified, it does not always exert a deleterious effect on the protein [11]. Nonetheless, we were able to register functional and structural disturbances on GlADI after exposing it to the assayed thiol-reactive drugs and compounds. The fluorescence emission spectra of GlADI showed both a decrease of 67% in quantum yield at an excitation wavelength of 295 nm and a blue shift of 6 nm when exposed to rabeprazole. This fluorescence decrease could implicate potential conformational changes in the protein structure, which would expose tryptophan residues to the milieu [28,29], probably weakening the stability of the GlADI structure. On the other hand, the blue shift is suggestive of a compaction effect on its 3D structure that could be impair the interactions of GlADI with other molecular elements; either with those of the microorganism itself or those of the host.

Drug repurposing, defined as finding new indications for existing drugs [30], is of particular interest for coping with giardiasis. Compared with developing drugs de novo, which is of very high cost (10 to 17 years and 800 million USD), drug repurposing significantly reduces both the time and money needed. However, repurposable drugs also have to succeed at a cellular level. In line with this, we found that of the four computationally identified drugs, only omeprazole and rabeprazole were able to efficiently inhibit cellular GlADI and exert a cytotoxic effect on trophozoites. Despite having succeeded in inhibiting GlADI in vitro, sulbutiamine and aurothiomalate were less effective against the cellular enzyme. They did not cause more than 40% inhibition of cellular GlADI activity, and they only slightly impacted the viability of trophozoites.

Sulbutiamine is a molecule consisting of two thiamin (B1vitamin) molecules bound together by a disulfide and is a lipophilic drug that is useful in alleviating fatigue [31]. Sulbutiamine specifically inactivates a glycolytic enzyme of *Encephalitozoon intestinalis* through interaction with cysteine residues [32], but it has not been tested in vitro against the parasites. Aurothiomalate exhibits anti-inflammatory properties, mediated through its reactivity with protein sulfhydryl groups [33]. Despite inhibiting recombinant GlADI, sulbutiamine and aurothiomalate were unable to inhibit cellular GlADI and effectively induce trophozoite death. These drugs are possibly inefficient for crossing the cell membrane of the trophozoites, or they could decrease the giardicidal activity by interacting with other molecules before reaching GlADI. This highlights the importance of corroborating the tests done with molecular targets in their cellular context.

In light of these findings, we focused on the capacity of GlADI as a druggable target for giardiasis using rabeprazole as an efficient repurposable drug. Our results show that encystment is impaired when a GlADI inhibitor such as rabeprazole is used. This could be due to the fact that *Giardia* uses ammonia from arginine metabolism to drive the synthesis of glucosamine-6-phosphate for cyst wall polysaccharide biosynthesis [34]. Moreover, the trophozoites treated with rabeprazole lack an essential source of ammonia, which might impair their capacity to produce the cyst wall. The use of rabeprazole might therefore help to ameliorate the dissemination of giardiasis by altering the cyst production. To the best of our knowledge, this is the first study where rabeprazole demonstrated its ability to target the encystment process of *Giardia*.

## 4. Materials and Methods

### 4.1. Reagents and Drugs

All reagents were purchased from Sigma–Aldrich (St. Louis, MO, USA) unless otherwise specified. Isopropyl-β-D-thiogalactopyranoside (IPTG) was from AMRESCO LLC (Cochran Road Solon, OH, USA); reduced nicotinamide adenine dinucleotide (NADH) was purchased from Roche (Penzberg, Upper Bavaria, Germany); Immobilized Metal Affinity Chromatography (IMAC) resin was purchased from Bio-Rad (Hercules, CA, USA); and Amicon Ultra 30 kDa filters were from Millipore Corporation (Billerica, MA, USA). Sulbutiamine was purchased from Santa Cruz, Biotechnology (Dallas, TX, USA).

### 4.2. Homology Modeling

In a previous work [18], two homology models based on the crystallographic structures of arginine deiminase from *Pseudomonas aeruginosa* and *M. arginini* were found to be the most appropriate templates for the modeling of 3D GlADI structure. Based on its described homodimer quaternary structure, ADI from *M. arginini* was selected as a template. The amino acid sequence of GlADI (NCBI reference: XP_001705755) [34] was obtained from the NCBI database. To build the three-dimensional model, the complete sequence of 580 amino acids was submitted to SWISS-MODEL of the Expasy Server (https://swissmodel.expasy.org/ (accessed on 11 November 2018)), the crystallographic coordinates from *M. arginini* ADI (Protein Data Bank code 1S9R [35]) were uploaded as a template. We used the VMD molecular graphics program to show the distribution of cysteine residues for the total GlADI dimer and show the catalytic residues’ distribution [36].

### 4.3. Docking

For docking studies, SwissDock server (http://www.swissdock.ch/docking (accessed on 5 March 2020)) was used, and the structure of the homology model of GlADI was analyzed as target, with s-methyl methanethiosulfonate (MMTS), dithio-bis-nitrobenzoic acid (DTNB), omeprazole, rabeprazole, sulbutiamine, and aurothiomalate as ligands. Ligands were obtained from ChemSpider (http://www.chemspider.com (accessed on 3 March 2020)) and PubChem Compound (https://www.ncbi.nlm.nih.gov/pccompound/ (accessed on 3 March 2020)) databases and adjusted to mol2 format with VMD. All structures were energy minimized with Chimera software [37], and with the resulting new coordinates, docking calculations were carried out with the mentioned server. The results were loaded and visualized with Chimera software [37].

### 4.4. Expression and Purification of Recombinant GlADI

The arginine deiminase gene from *Giardia lamblia* was cloned into the pET28a (+) vector (Novagen), as previously described [38]. pET28a (+) vector adds an N−terminal His6x tag to the protein. To over-express GlADI, this vector was used to transform the Rosetta 2 (DE3) pLysS *Escherichia coli* strain. This *E. coli* strain was grown in 250 mL of Terrific Broth culture medium supplemented with kanamycin and chloramphenicol at a final concentration of 50 μg/mL (each one). Cells were incubated at 37 °C until 0.8–1.0 of absorbance at 600 nm was reached. The overexpression was induced by the addition of isopropyl thio-β-Dgalactoside (IPTG) at a final concentration of 1 mM for 16 h at 16 °C. Cells were harvested by centrifugation at 2370× *g* at 12 °C for 5 min (pellets were stored at −80 °C until further use). Pellets were weighed and resuspended in 25 mL of lysis buffer containing 50 mM sodium phosphate monobasic (H_2_NaO_4_P), 300 mM sodium chloride (NaCl), 20% glycerol, 4 μM β-mercaptoethanol, lysozyme (1 μg/mL), and protease inhibitor cocktail (1 mL of stock solution per 20 g of wet weight of *E. coli* cells). The buffer was adjusted at pH 8.0. The bacterial suspension was disrupted by sonication and centrifuged at 7690× *g* for 1 h at 4 °C. Protein purification was performed by IMAC using a Profinity Ni^2+^ charged resin previously equilibrated with lysis buffer. The soluble protein fraction was passed through an IMAC column three times, and the recombinant GlADI was purified with a gradient of 20–250 mM imidazole. The purified protein was dialyzed against lysis buffer and concentrated to a volume of 250 μL using Amicon ultrafiltration units. Due to the low stability of GlADI in solution, the N-terminal His6x tag was not cleaved. The concentration of purified GlADI was spectrophotometrically determined (Spectrophotometer Cary 50, Agilent Technologies, Santa Clara, CA, USA) at 280 nm using the extinction coefficient of ε_280_ = 60,250 M^−1^ cm^−1^ [39]. The purity of the enzyme was analyzed by sodium dodecyl sulfate-polyacrylamide gel electrophoresis technique (16% SDS–PAGE) and stained with colloidal Coomassie Brilliant Blue.

### 4.5. Enzyme Activity Assays

The enzyme activity of GlADI was determined by monitoring the conversion of L-arginine to citrulline. The reaction was based on that reported by Weickmann [40] and modified by Li [19], with slight modifications by us. Briefly, the reaction mixture at pH 8.0 in buffer containing 50 mM sodium phosphate monobasic (H_2_NaO_4_P), 300 mM sodium chloride (NaCl), and 20% glycerol was added with 8 mM L-arginine, 10 mM α-ketoglutaric acid, 0.2 mg/mL glutamate dehydrogenase (GIDH) as coupling enzyme, and 0.2 mM NADH. The reaction was initialized by adding the recombinant enzyme or soluble extracts from trophozoites to the reaction mixture. Enzyme activity was spectrophotometrically measured by monitoring NADH oxidation at 340 nm, at 25 °C.

### 4.6. Enzyme Inactivation Assays of Recombinant GlADI and Second-Order Inactivation Constants

The sulfhydryl reagents, thiol-reactive drugs, and albendazole solutions were freshly prepared in lysis buffer before use, as previously described [11,32,41]. The GlADI inactivation assays were performed at a protein concentration of 0.5 mg/mL in lysis buffer for 2 h at 25 °C. Aliquots from these incubations were withdrawn and assayed for enzyme activity, as described above. The reaction was initiated by adding 17 μg/mL of GlADI to the reaction mixture. The data are reported as the percentage of residual activity. Results are from at least four technical repetitions.

Pseudo-first-order inactivation rate constants (*k*_1_) were obtained at MMTS concentrations ranging from 2.5 to 85 μM, whereas the concentration for rabeprazole varied from 5 to 110 μM, at the times indicated in Figure 4. Aliquots were removed from the samples and assayed for residual activity in the reaction mixture described above. Data were adjusted to a mono-exponential decay model, $A_{R}=A_{0}\;e^{-kt}$, where *A*_R_ is the residual activity at time *t*, *A*_0_ is the activity at time 0, and *k* is the pseudo-first-order inactivation rate constant [41]. The slopes of the linear plots (*k*_1_) versus the concentration of MMTS and rabeprazole correspond to the second-order inactivation constant *k*_2_ (M^−1^ s^−1^).

### 4.7. Fluorescence Emission Spectra

Fluorescence experiments were performed using a Perkin–Elmer LS55 spectrofluorometer at 25 °C and a protein concentration of 65 μg/ mL. The intrinsic fluorescence of the enzymes was determined at an excitation wavelength of 295 nm, and the emission spectra were recorded from 310 to 500 nm, with an integration time of 1 s, using excitation and emission slits of 3 nm. Each spectrum was the average of three scans with two experimental repetitions. The spectra of blanks were subtracted from those containing protein.

### 4.8. Quantification of Free Cysteines after Drug Treatments

The Cys residues were determined using Ellman’s reagent under non-denaturing and denaturing conditions [42]. To carry out the experiments, 0.5 mg/mL of GlADI was incubated, either without compounds or with 250 µM DTNB and MMTS, separately, or with 750 µM omeprazole, rabeprazole, sulbutiamine, or aurothiomalate, separately. Each mix was incubated for 2 h at 25 °C. After the incubation period, the samples were extensively washed by ultrafiltration with Centricon filters to eliminate the excess drug, and the protein concentration was estimated by determining the absorbance at 280 nm. Next, the free Cys content of the samples was spectrophotometrically quantified as follows: the basal absorbance of 1 mM DTNB and 5% SDS dissolved in lysis buffer was measured at 412 nm (ε 412 nm = 13.6 mM/cm), and the increase in absorbance following the addition of 200 µg of protein was monitored. The number of modified Cys residues was indirectly calculated by subtracting the number of free Cys residues in the derivatized enzyme (treated with the drugs) from the number of free Cys residues in the enzyme in the absence of the drugs. The results represent the mean ± SD of at least four independent experiments.

### 4.9. Drug Susceptibility of Trophozoites and Cellular GlADI Activity Assays

*G. lamblia* WB strain was acquired from the American Type Culture Collection (ATCC) and cultured, harvested, and maintained as previously described [27]. Trophozoites were cultured in the presence of increasing concentrations of omeprazole, rabeprazole, sulbutiamine, or aurothiomalate for 24 h at 37 °C, at the concentrations shown in Figure 5. At the end of the treatment period, the tubes were decanted to preserve only the adhered trophozoites (trying to keep only living cells, and without mixing with dead cells). Nonetheless, the living and dead trophozoites were counted from these supernatants to register the total viability after the treatments. The culture tubes were washed three times with phosphate-buffered saline (PBS), pH 7.4, and chilled on ice for 20 min, and then the trophozoites were counted to standardize the assays by the number of cells. The cells were disrupted with five cycles of freeze–thaw cycles in liquid N_2_ and 42 °C, in lysis buffer. Disrupted cells were centrifuged at 15,700× *g* for 20 min at 4 °C, and the supernatants were withdrawn for analysis of residual GlADI activity using the coupled method mentioned above, at 25 °C. We considered 100% to be the enzyme activity registered for the cultures without drugs.

### 4.10. Induction to Encystment in the Presence of Rabeprazole

*G. lamblia* WB strain trophozoites were cultured as mentioned above and harvested in the exponential phase of growth (~2 × 10^6^ cells/mL). Encystation was accomplished following the method described by Boucher and Gillin with modifications [43]. Briefly, trophozoites were counted in a hemocytometer chamber and added to pre-encystation medium containing TYI-S-33 growth medium (pH 7.1) with the antibiotics ceftazidime (500 μg/mL; PiSA Laboratories) and ampicillin (500 μg/mL; Sigma-Aldrich, St. Louis, MO, USA), but no bovine bile. The culture tubes (borosilicate glass screw-capped tubes of 8 mL) were divided into 2 groups of three tubes each per assay. The experimental group was supplemented with 150 μM rabeprazole. Pre-encystation cultures were grown for 24 h at 37 °C. The tubes were inverted eight times, and the unattached trophozoites and medium were discarded. The attached trophozoite monolayers were refed with fresh encystation medium consisted of pre-encystation medium adjusted to pH 8.0 with 1 M NaOH and supplemented with bovine bile (7.5 mg/mL, final concentration) and lactic acid (hemi-calcium salt, 5 mM, final concentration), and grown for 48 h at 37 °C. The experimental group was kept in a 150 μM rabeprazole condition. Parasites were harvested and counted to report the number of trophozoites and cysts per group.

### 4.11. Statistical Analyses

Data were analyzed with GraphPad Prism™ software (version 8.2.1 for Windows™, San Diego, CA, USA). The normal distribution of data was assessed with the Shapiro–Wilk test. Normally distributed data were analyzed with one-way ANOVA, followed by Dunnett’s multiple comparisons adjustment. Non-parametric distributed data were analyzed with the Mann–Whitney U test or Friedman test, followed by Dunn’s multiple comparisons adjustment test. Results are expressed as mean ± SD or as the median and interquartile range (IQR) for data with parametric and non-parametric distribution, respectively. A *p*-value < 0.05 was considered to be statistically significant.

## Figures and Tables

**Table 1:** Cys residues with more than 10% ASA.

Cys Position	ASA (%)	Chain
329	71.3/46.5	A/B
396	66.2/31.4	A/B
300	63.3/58.1	A/B
374	24.1/28.0	A/B
271	25.1	A
223	21.1	B
118	16.4/15.8	A/B
216	15.4	B
52	13.7	A

**Table 2:** Number of modified Cys residues per monomer.

Giardial Arginine Deiminase (GlADI)	Number of Modified Cys/Monomer
+DTNB (250 µM)	6 ± 0.1
+MMTS (250 µM)	6 ± 0.3
+Omeprazole (750 µM)	2 ± 0.9
+Rabeprazole (750 µM)	5 ± 0.3
+Sulbutiamine (750 µM)	2 ± 0.9
+Aurothiomalate (750 µM)	5 ± 0.5

Untreated GlADI shows 9 ± 0.9 free Cys/monomers. GlADI treated with 1mM TCEP shows 16 ± 0.5 free Cys/monomers.

## Data Availability

Data is contained within the article.

## Supplementary Materials

The following are available online at https://www.mdpi.com/article/10.3390/ijms22179491/s1, Figure S1: Studied molecules 2D chemical structure, Figure S2: Multiple sequence amino acid alignment of ADI enzyme from *G. lamblia* and different organisms.

## Abbreviations

DTNB	Dithio-bis-nitrobenzoic acid
MMTS	S-methyl methanethiosulfonate
GlADI	Arginine deiminase from *G. lamblia*
PPI	Proton pump inhibitor
ATP	Adenosine triphosphate
ASA	Accessible surface area
Cys	Cysteine
a.u.	Arbitrary units
*k*_i_	Pseudo-first-order inactivation rate constant
*k*_2_	Second-order rate constant
TCEP	Tris(2-carboxyethyl)phosphine
SD	Standard deviation
SDS	Sodium dodecyl sulfate
ATCC	American Type Culture Collection
PBS	Phosphate-buffered saline
