# Supplementary material for: Multilevel Approach for the Treatment of Giardiasis by Targeting Arginine Deiminase
Source: Int J Mol Sci. 2021 Aug 31;22(17):9491. doi: 10.3390/ijms22179491 (PMC8431557; doi:10.3390/ijms22179491)
Supplement: Supplementary file 1 [file ijms-22-09491-s001.zip › ijms-1311862-supplementary.pdf]

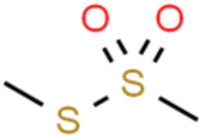

MMTS
PubChem CID: 18064

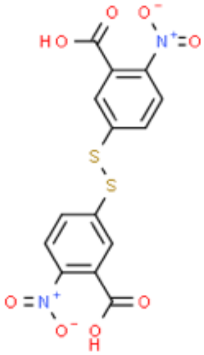

DTNB
PubChem CID: 6254

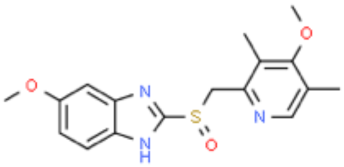

Omeprazole
PubChem CID: 4594

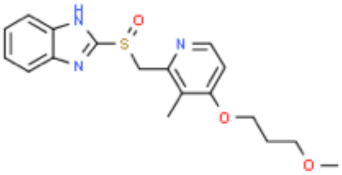

Rabeprazole
PubChem CID: 5029

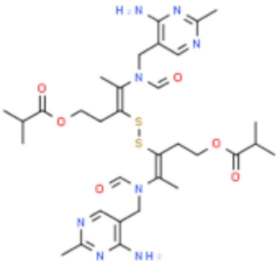

Sulbutiamine
PubChem CID: 20055424

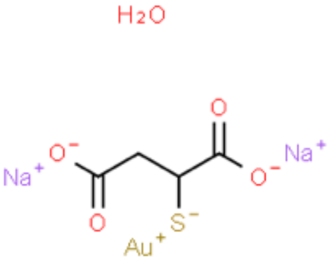

Aurothiomalate
PubChem CID: 134577

Figure S1. Studied molecules 2D chemical structure. The structures were obtanied from Chemspider free chemical structure database (www.chemspider.com).

```
XP_001705755.1  MTDFSKDKEKLAQATQGGENERAEIVVVHLPQGTS-FLTSLNPEGNLLEEPICPDELRRD    59
EST48861.1      ---------MQIKPQQHSEHATARTILVYEPELAV-FVNCLHPAGSLYECPINPESALAQ    50
WP_011027247.1  --MTSNESAVSQALGVHSEVGRLRKVLVCSPGLAHRRLTPTNSDELLFDDVMWVENAQRD    58
WP_012482189.1  ----------MRGFGVHSEVGKLRTVMVCRPSLAHQRLTPANCHDLLFDDVLWVHEAQKD    50
KFL10416.1      ------MSTEKTKLGVHSEAGKLRKVMVCSPGLAHQRLTPSNCDELLFDDVIWVNQAKRD    54
KHL72821.1      ------MSAEKQKYGVHSEAGKLRKVMVCAPGLAHKRLTPSNCDELLFDDVIWVDQAKRD    54
AAD55448.1      ---MSVFDSKFKGIHVYSEIGELETVLVHEPGKEIDYITPARLDELLFSAILESHDARKE    57
BAQ54605.1      ---MSVFDSKFKGIHVYSEIGELESVLVHEPGREIDYITPARLDELLFSAILESHDARKE    57
AGS66837.1      -----MEEEYLNPINIFSEIGRLKKVLLHRPGEELENLTPLIMKNFLFDDIPYLKVARQE    55
NP_813907.1     ---------MSHPINVFSEIGKLKTVMLHRPGKELENLMPDYLERLLFDDIPFLEKAQAE    51
ARW56296.1      ---------MTTPIHVYSEIGPLKTVMLKRPGRELENLTPEYLERLLFDDIPFLPAVQKE    51
CAA04682.1      ---------MTSPIHVNSEIGKLKTVLLKRPGKEVENITPDIMYRLLFDDIPYLPTIQKE    51
                                 .*    . :::  *      :        * .          :

XP_001705755.1  HEGFQAVLKEKGCRVYMPYDVLSEAS--P--AEREVLMDQAMASLKYELHATGARITPKM    115
EST48861.1      HRNFQKVLQSHGINVLEVCEILRSNTTDP--AQRLELEEYAAQQLTY----------IGP    98
WP_011027247.1  HAAFVGELRRRGVEVVELHDLLAQIMALP--EARAWLLDRKITANQ-----VGIGL----    107
WP_012482189.1  HFDFVLKMQERGVEVLELHDLLGQTLANQ--QARDFLLDRRITPNV-----LGSQI----    99
KFL10416.1      HFDFVTKMRERGIDVLEMHNLLTETIQNP--EALKWILDRKITADS-----VGLGL----    103
KHL72821.1      HFDFVTKMRERGVDVLEMHNLLTDIVQNK--DALKWILDRKITPDT-----VGVGL----    103
AAD55448.1      HKEFVAELKKRGINVVELVDLIVETYDLASKEAKEKLLEEFLDDSAP---VLSDEH----    110
BAQ54605.1      HKQFVAELKANDINVVELIDLVAETYDLASQEAKDKLIEEFLEDSEP---VLSEEH----    110
AGS66837.1      HEVFVNILKDNSVEIEYVEDLVSEVLASS-VALKNKFISQFILEAEI----KTDGV----    106
NP_813907.1     HDAFAELLRSKDIEVVYLEDLAAEALINE--EVRRQFIDQFLEEANI----RSESA----    101
ARW56296.1      HDQFAETLKQQGAEVLYLEKLTAEALDDA--LVREQFIDELLTESKA----DINGA----    101
CAA04682.1      HDQFAQTLRDNGVEVLYLENLAAEAIDAG--DVKEAFLDKMLNESHI----KSPQV----    101
                *  *   :: ..  :    .:  .            :  .

XP_001705755.1  KYCVSDEYKRKVLSALST-RNLVDVILSEPVIHLAPGVRNT--------ALVTNSVEIHD    166
EST48861.1      EEYASDDYKMKVIKSLSP-ESLVKVILTAPVVYLEATTNNT--------NVITTKTEMRA    149
WP_011027247.1  -----IDAARAYLETLSP-RELAEYLVGGLATSDLPEDFRSPHLALARESTGAREYLMPP    161
WP_012482189.1  -----AEAMRPWLDEMPA-AQLAAFMIGGIAISDLPEVRAKSL---MLSALPETDFVIPP    150
KFL10416.1      -----TSELRSWLESLEP-RKLAEYLIGGVAADDLPASEGADILKMYREYLGHSSFLLPP    157
KHL72821.1      -----TNEVRSWLEGLEP-RHLAEFLIGGVAGQDLPQSEGADVVKMYNDYLGHSSFILPP    157
AAD55448.1      -----RAAVKKFLQSQKSTRSLVEYMIAGITKHDLKI-------------ESDLELIVDP    152
BAQ54605.1      -----KVVVRNFLKAKKTSRELVEIMMAGITKYDLGI-------------EADHELIVDP    152
AGS66837.1      -----INILKDYFSNLTV-DNMVSKMISGVAREELKDCEF-SLDDWV---NGSSLFVIDP    156
NP_813907.1     -----KEKVRELMLEIDDNEELIQKAIAGIQKQELPKYEQEFLTDMV---EADYPFIIDP    153
ARW56296.1      -----YDRLKEFLLTFDA-DSMVEQVMSGIRKNELEREKKSHLHELM---EDHYPFYLDP    152
CAA04682.1      -----QAALKDYLISMAT-LDMVEKIMAGVRTNEIDIKSKALIDVSA---DDDYPFYMDP    152
                            :        :    :                              :

XP_001705755.1  SNNMVFMRDQQITTRRGIVMGQFQAPQRRREQVLALIFWKRLGARVVGD-----------    215
EST48861.1      LGNIVFTRDQQITTAKGVVIGNFSAEQRLGENALMEFVLKKIGIRPIGRIGEFTYERHIP    209
WP_011027247.1  LPNTLYTRDTTCWLYGGLTLNPLYWSARHDETLLMKAIYTFHPDFK-GS--KVWW-----    213
WP_012482189.1  IPNTLFQRDPSCWIYGGVTCNPMFWPARRAETLIQRAIYKFHPIFL-GGDFQIWW-----    204
KFL10416.1      LPNTQFTRDTTCWIYGGVTLNPMYWPARRQETLLTTAIYKFHPEFA-NAEFEIWY-----    211
KHL72821.1      LPNTQFTRDTTCWIYGGVTLNPMYWPARRQETLLTTAIYKFHPQFT-GADFQVWY-----    211
AAD55448.1      MPNLYFTRDPFASVGNGVTIHYMRYKVRQRETLFSRFVFSNHPKLV---NTPWYY-----    204
BAQ54605.1      MPNLYFTRDPFASVGNGVTIHYMRYKVRQRETLFSRFVFSNHPKLI---NTPWYY-----    204
AGS66837.1      MPNVLFTRDPFASIGNGITINKMYTKVRRRETIFAEYIFKYHSAYK--ENVPIWF-----    209
NP_813907.1     MPNLYFTRDNFATMGHGISLNHMYSVTRQRETIFGQYIFDYHPRFA-GKEVPRVY-----    207
ARW56296.1      MPNLYFTRDPAAAIGSGLTINKMKEPARRRESLFMRYIINHHPRFK-GHEIPVWL-----    206
CAA04682.1      MPNLYFTRDPAASMGDGLTINKMTFEARQRESMFMEVIMQHHPRFA-NQGAQVWR-----    206
                  *  : **        *:     :    *  *  :   .

XP_001705755.1  ----CREGGPHCMLEGGDFVPVSPGLAMMGVGLRSTYVGAQYLMSKDLLGTR--RF-AVV    268
EST48861.1      ICGGANKLTPKATIEGGDFIALNNGIGALGVGLRSSYTAGIHLMEHDLLGTD--KF-IIV    266
WP_011027247.1  --GDPERDWGQATFEGGDIMPVGNGVVLMGMSERTSRQAITQV-AAALFRSG--AAEHVV    268
WP_012482189.1  --GDSDQPFANASMEGGDVMPIGNGTVLIGMGERTTYQAVGQV-AQALFRSK--AAKRVI    259
KFL10416.1      --GDPDKDHGSSTLEGGDVMPIGNGVVLIGMGERSSRQAIGQV-AQSLFAKG--AAERVI    266
KHL72821.1      --GDPDKDHGNATLEGGDVMPIGKGIVLIGMGERTSRQAIGQL-AQNLFAKG--AVEKVI    266
AAD55448.1      --DPA----EGLSIEGGDVFIYNNDTLVVGVSERTDLQTITLL-AKNIKANKECEFKRIV    257
BAQ54605.1      --DPS----LKLSIEGGDVFIYNNDTLVVGVSERTDLQTVTLL-AKNIVANKECEFKRIV    257
AGS66837.1      --NRW----EETSLEGGDEFVLNKDLLVIGISERTEAGSVEKL-AASLFKNK-APFSTIL    261
NP_813907.1     --DRS----ESTRIEGGDELILSKEVVAIGISQRTDAASIEKI-ARNIFEQK-LGFKNIL    259
ARW56296.1      --DRD----FKFNIEGGDELVLNEETVAIGVSERTTAQAIERL-VRNLFQRQ-SRIRRVL    258
CAA04682.1      --DRD----HIDRMEGGDELILSDKVLAIGISQRTSAQSIEEL-AKVLFANH-SGFEKIL    258
                             :**** .  .     :*:. *:       :    :          ::

XP_001705755.1  KDCFDQHQDRMHLDCTFSVLHDKLVVLDDYI-CSGMGLRYVDEWID-VGAD--A----VK    320
```

```
EST48861.1          KDTFDQDQDRMHLDCTCSPLHDKLILIDEEILINPDKLRYVDEWVH-LGGY--S----DE    319
WP_011027247.1      VAGMPKLRSAMHLDTVFTFADRDVVTLYPRIMDAV---HTFSLRPGDRAPGFDVV--DEG    323
WP_012482189.1      GCLMPKSRAAMHLDTVFSFCDRDVVTLFAEVVDQV---RCYSMYPKDDD-GTFEI--HPE    313
KFL10416.1          VAGLPKSRAAMHLDTVFSFCDRDLVTVFPEVVKEI---VPFSLRPDPSSPYGMNI--RRE    321
KHL72821.1          VAGLPKSRAAMHLDTVFSFCDRDLVTIFPEVVKEI---VPFIIRPDESKPYGMDV--RRE    321
AAD55448.1          AINVPKWTNLMHLDTWLTMLDKDKFLYSPIANDVF---K-FWDYDLVNG--GDAPQPVDN    311
BAQ54605.1          AINVPKWTNLMHLDTWLTMLDKDKFLYSPIANDVF---K-FWDYDLVNG--GAEPQPVEN    311
AGS66837.1          AFKIPKNRAYMHLDTVFTQIDYSVFTSFTS---DD---MYFSIYVLTYNSNSNKINIKKE    315
NP_813907.1         AFDIGEHRKFMHLDTVFTMIDYDKFTIHPEIEGGL---V---VYSITEKADGDIQIT-KE    312
ARW56296.1          AVEIPKSRAFMHLDTVFTMVDRDQFTIHPAIQGPE---GDMRIFVLERGKTADEIHT-TE    314
CAA04682.1          AIKIPHKHAMMHLDTVFTMIDYDKFTIHPGIQGAG---GMVDTYILEPGNN-DEIKI-TH    313
                       . .    ****   :  . . .

XP_001705755.1      KAKSSAVTCGNYVLAKAN------------VEFQQWLSENGYTIVRIPHEYQLAYGCNNL    368
EST48861.1          HKSFY-----ALNEKRTN------------IELKQYLEQSGYSIIAIPHKYQLAYGCNML    362
WP_011027247.1      STPFVDVVAKALGLPKLR--VVETGGDAYASERQQWDSGNNA--VAVEPGVVFTYDRNT-    378
WP_012482189.1      NRPMLDVVAEALDLPQLR--TVETGGNSYQAEREQWDDGNNV--VALEPGVVVAYDRNT-    368
KFL10416.1          EKTFLEVVAESLGLKKLR--VVETGGNSFAAEREQWDDGNNV--VCLEPGVVVGYDRNT-    376
KHL72821.1          NKSFIEVVGEQLGV-KLR--VVETGGNSFAAEREQWDDGNNV--VAVEPGVVIGYDRNT-    375
AAD55448.1          GLPLEDLLKSIIGKKPTLIPIAGAGASQIDIERETHFDGTNY--LAVAPGIVIGYARNE-    368
BAQ54605.1          GLPLEGLLQSIINKKPVLIPIAGEGASQMEIERETHFDGTNY--LAIRPGVVIGYSRNE-    368
AGS66837.1          KAKLKDVLSFYLG-RKID-IIKCAGGDLIHGAREQWNDGANV--LAIAPGEVIAYSRNH-    370
NP_813907.1         KDTLDNILCKYLHLDNVQ-LIRCGAGNLTAAAREQWNDGSNT--LAIAPGEVVVYDRNT-    368
ARW56296.1          EHNLPEVLKRTLGLSDVN-LIFCGGGDEIASAREQWNDGSNT--LAIAPGVVVTYDRNY-    370
CAA04682.1          QTDLEKVLRDALEVPELT-LIPCGGGDAVVAPREQWNDGSNT--LAIAPGVVVTYDRNY-    369
                                                     :   .  .   : :     . *  *

XP_001705755.1      NLGN----NCVLSVHQPTVDFIKADPAYISYCKSNNLPNGLDLVYVPFRGITRMYGSLHC    424
EST48861.1          HLGFTDGAFHILTVHEESKLFIERHPVFKTYCQKNNIR--IVVEYVPFRSITSMYGSLHC    420
WP_011027247.1      ------------------------------LTNALLREARVEVVTIVGAELGRGRGGGHC    408
WP_012482189.1      ------------------------------YTNTLLRKAGIEVITIRGSELGRGRGGGHC    398
KFL10416.1          ------------------------------YTNTLLRKAGVEVITISASELGRGRGGGHC    406
KHL72821.1          ------------------------------YTNTLLRKAGIEVITISAGELGRGRGGGHC    405
AAD55448.1          ------------------------------KTNAALEAAGITVLPFRGNQLSLGMGNARC    398
BAQ54605.1          ------------------------------KTNAALEAAGIKVLPFHGNQLSLGMGNARC    398
AGS66837.1          ------------------------------VTNKLFEENGIKVHRIPSSELSRGRGGPRC    400
NP_813907.1         ------------------------------ITNKALEEAGVKLNYIPGSELVRGRGGPRC    398
ARW56296.1          ------------------------------ISNECLREQGIKVIEIPSGELSRGRGGPRC    400
CAA04682.1          ------------------------------VSNENLRQYGIKVIEVPSSELSRGRGGPRC    399
                                                    :       : :  .    :    *. :*

XP_001705755.1      ASQVVYRTPLAPAAVKACEQEGDGIAAIYEKNGEPVDAAGKKFDCVIYIPSSVDDLIDGL    484
EST48861.1          ATQVLERDVP-TAAIDKCNS--------------QVLNDEKLFDAALFIPTFF-------    458
WP_011027247.1      MTCPLVREPVDF------------------------------------------------    420
WP_012482189.1      MTCPIWRDPAY-------------------------------------------------    409
KFL10416.1          MTCPIIRDPIDY------------------------------------------------    418
KHL72821.1          MTCPIVRDPIDY------------------------------------------------    417
AAD55448.1          MSMPLSRKDVK-------------------------------------------------    409
BAQ54605.1          MSMPLSRKDVKW------------------------------------------------    410
AGS66837.1          MSMSLVREDI--------------------------------------------------    410
NP_813907.1         MSMPLYREDL--------------------------------------------------    408
ARW56296.1          MSMPLYREDVK-------------------------------------------------    411
CAA04682.1          MSMPLVRRKT--------------------------------------------------    409
                     :  : *

XP_001705755.1      KINLRDDAAPSREIIADAYGLYQKLVSEGRVPYITWRMPSMPVVSLKGAA---KAGSLKA    541
EST48861.1          ---------ASKETIKEVYELYEKLRKEGKNVYLINKYKITELISFKNLNAKNVEGLLAE    509
WP_011027247.1      ------------------------------------------------------------    420
WP_012482189.1      ------------------------------------------------------------    409
KFL10416.1          ------------------------------------------------------------    418
KHL72821.1          ------------------------------------------------------------    417
AAD55448.1          ------------------------------------------------------------    409
BAQ54605.1          ------------------------------------------------------------    410
AGS66837.1          ------------------------------------------------------------    410
NP_813907.1         ------------------------------------------------------------    408
ARW56296.1          ------------------------------------------------------------    411
CAA04682.1          ------------------------------------------------------------    409

XP_001705755.1      VLDKIPQLTPFTPKAVEGAPAAYTRYLGLEQADICVDIK        580
EST48861.1          IYGKSAKKHFIQPKKQDGAVTAIER-DGLSLDDILYIF-        546
```

```
WP_011027247.1      ---------------------------------------      420
WP_012482189.1      ---------------------------------------      409
KFL10416.1          ---------------------------------------      418
KHL72821.1          ---------------------------------------      417
AAD55448.1          ---------------------------------------      409
BAQ54605.1          ---------------------------------------      410
AGS66837.1          ---------------------------------------      410
NP_813907.1         ---------------------------------------      408
ARW56296.1          ---------------------------------------      411
CAA04682.1          ---------------------------------------      409
```

Figure S2. Multiple sequence amino acid alignment of ADI enzyme from *G. lamblia* and different organisms. The alignment was performed in the free tool Clustal, version 1.2.4. The reference sequences used are: XP_001705755.1 Arginine deiminase [*Giardia lamblia* ATCC 50803]. EST48861.1 Arginine deiminase [*Spironucleus salmonicida*]. WP_011027247.1 Arginine deiminase [*Streptomyces coelicolor*]. WP_012482189.1 Arginine deiminase [*Rhizobium etli*]. KFL10416.1 Arginine deiminase [*Pseudomonas aeruginosa*]. KHL72821.1 Arginine deiminase [*Pseudomonas putida*]. AAD55448.1 Arginine deiminase [*Mycoplasma arthritidis*]. BAQ54605.1 Arginine deiminase [*Mycoplasma arginini*]. AGS66837.1 Arginine deiminase [*Borrelia burgdorferi* CA382]. NP_813907.1 Arginine deiminase [*Enterococcus faecalis* V583]. ARW56296.1 Arginine deiminase [*Bacillus licheniformis*]. CAA04682.1 Arginine deiminase [*Lactobacillus sakei*]. Symbols: * (asterisk) indicates positions which have a single, fully conserved residue. : (colon) indicates conservation between groups of strongly similar properties. . (period) indicates conservation between groups of weakly similar properties. – (hyphen) indicates a gap. Cysteine residues are highlighted in bold red. The C-terminal end of the protein is highlighted in bold. Residues of the catalytic site (Glu 226, His 280, and Cys 424) are highlighted in blue.
